# Gut-bone axis research: unveiling the impact of gut microbiota on postmenopausal osteoporosis and osteoclasts through Mendelian randomization

**DOI:** 10.3389/fendo.2024.1419566

**Published:** 2024-05-31

**Authors:** Hefang Xiao, Yaobin Wang, Yi Chen, Rongjin Chen, Chenhui Yang, Bin Geng, Yayi Xia

**Affiliations:** ^1^Department of Orthopaedics, Lanzhou University Second Hospital, Lanzhou, China; ^2^Gansu Province Orthopaedic Clinical Medicine Research Center, Lanzhou, China; ^3^Gansu Province Intelligent Orthopedics Industry Technology Center, Lanzhou, China

**Keywords:** gut microbiota, postmenopausal osteoporosis, osteoclasts, Mendelian randomization, gut-bone axis, GWAS

## Abstract

**Background:**

Postmenopausal osteoporosis is a prevalent disease that affects the bone health of middle-aged and elderly women. The link between gut microbiota and bone health, known as the gut-bone axis, has garnered widespread attention.

**Methods:**

We employed a two-sample Mendelian randomization approach to assess the associations between gut microbiota with osteoclasts and postmenopausal osteoporosis, respectively. Single nucleotide polymorphisms associated with the composition of gut microbiota were used as instrumental variables. By analyzing large-scale multi-ethnic GWAS data from the international MiBioGen consortium, and combining data from the eQTLGen consortium and the GEFOS consortium, we identified microbiota related to osteoclasts and postmenopausal osteoporosis. Key genes were further identified through MAGMA analysis, and validation was performed using single-cell data GSE147287.

**Results:**

The outcomes of this study have uncovered significant associations within the gut microbiome community, particularly with the Burkholderiales order, which correlates with both an increase in osteoclasts and a reduced risk of postmenopausal osteoporosis. with an odds ratio (OR) of 0.400, and a P-value of 0.011. Further analysis using single-cell data allowed us to identify two key genes, FMNL2 and SRBD1, that are closely linked to both osteoclasts and osteoporosis.

**Conclusion:**

This study utilizing Mendelian randomization and single-cell data analysis, provides new evidence of a causal relationship between gut microbiota and osteoclasts, as well as postmenopausal osteoporosis. It was discovered that the specific microbial group, the Burkholderiales order, significantly impacts both osteoporosis and osteoclasts. Additionally, key genes FMNL2 and SRBD1 were identified, offering new therapeutic strategies for the treatment of postmenopausal osteoporosis.

## 1 Introduction

Postmenopausal osteoporosis (PMOP) is a systemic skeletal condition characterized by reduced bone mass, microarchitectural deterioration of bone tissue, increased bone fragility, and susceptibility to fracture due to estrogen deficiency following menopause. Primarily affecting middle-aged and elderly women, it stands as a significant factor contributing to their elevated risk of fractures. The diagnosis of osteoporosis hinges on bone density measurements, predominantly through Dual-energy X-ray Absorptiometry (DEXA) scanning, which precisely gauges bone density at the spine and hip (1). Epidemiological studies underscore that the incidence of osteoporosis escalates with age among postmenopausal women. Globally, over one-third of women aged 50 and above are likely to experience at least one fracture due to osteoporosis during their remaining lifetime. Fractures not only heighten the risk of mortality but also severely impair the quality of life and independence of patients, imposing significant societal and familial burdens. Consequently, the prevention and management of osteoporosis have emerged as pressing public health concerns globally (2).

The gut microbiota, a complex and vast community of microorganisms residing in the human gastrointestinal tract, has been shown to play a crucial role in bone metabolism and health through various mechanisms involving the immune system, metabolic products, and the endocrine system (3). These insights have illuminated potential therapeutic targets for osteoporosis treatment, including modulation of the gut microbiome composition or its metabolic products to enhance bone health. The interplay between the gut microbiota and bone health has garnered increasing attention, revealing the microbiota’s involvement in digestion, metabolism, immune function, and its impact on bone metabolism and health through diverse mechanisms (4). Research has discovered that the gut microbiota modulates immune system function, thereby affecting bone metabolism and regulating bone density. Studies on germ-free mouse models indicate that mice devoid of gut microbiota exhibit increased bone density. This observation suggests a significant role for the gut microbiota in regulating bone metabolism. The absence of gut microbiota appears to influence bone resorption and formation processes, potentially through interactions with the immune system. Specifically, gut microbiota may modulate the activity of osteoclasts (cells that break down bone tissue) and osteoblasts (cells that form new bone tissue) by affecting immune responses. Consequently, the presence of gut microbiota is essential for maintaining a balance between bone resorption and formation, which is crucial for bone health (5). Many investigations have also explored how gut microbiota affects bone metabolism through various mechanisms, including the modulation of host metabolism, immune responses, and hormone secretion (6). This encompasses the microbiota’s impact on nutrient absorption, the gut-brain axis, and its immunomodulatory effects on the balance between osteoblasts and osteoclasts (7). Research indicates that gut microbiota influence bone metabolism through various mechanisms, including the regulation of host metabolism, immune responses, and hormone secretion. These include the microbiota’s impact on nutrient absorption, the gut-brain axis, and its immunomodulatory effects on the balance between osteoblasts and osteoclasts. For example, gut microbiota influence the host’s immune system by modulating bile acid metabolism, particularly by regulating colonic FOXP3+ regulatory T cells, which is crucial for protecting against inflammatory colitis (8). Additionally, gut microbiota affect the host’s intestinal immune balance through tryptophan metabolism, where tryptophan and its metabolites, such as indole and serotonin, regulate intestinal immunity and microbiota stability (9). Moreover, gut microbiota regulate bone metabolism by influencing the immune system, potentially impacting bone density and bone metabolism, suggesting gut microbiota as a novel therapeutic target for the treatment of osteoporosis and fracture prevention (3).

**Figure 1:**
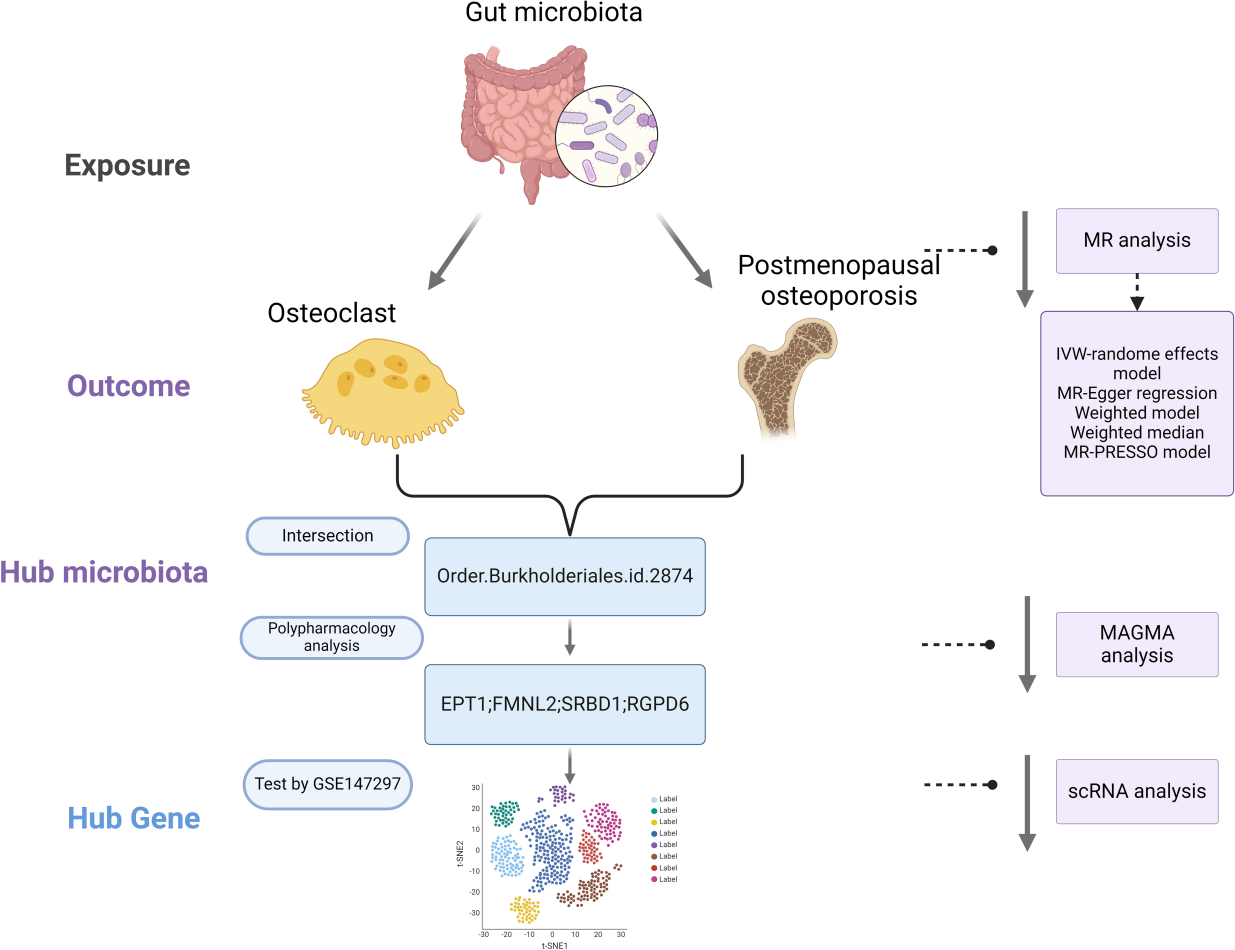
Workflow of our research.

Mendelian Randomization (MR) is a method that employs genetic variants as instrumental variables to assess the causal impact of exposure factors on outcomes. By leveraging the random allocation of genetic variants, MR can mitigate confounding and reverse causation issues inherent in observational studies, offering more robust causal inferences. MR has found successful applications across various domains, including cardiovascular diseases, metabolic disorders, and nutrition research (10). The advent of large-scale Genome-Wide Association Studies (GWAS) and Mendelian Randomization (MR) has enabled the assessment of causal relationships between the gut microbiota, osteoclasts, and postmenopausal osteoporosis. This study utilizes publicly available summary data from GWAS (11). In the initial phase, we investigate the causal effects of gut microbiota on osteoclasts. Subsequently, we explore the causal relationship between gut microbiota and postmenopausal osteoporosis. In the final phase, we identify the interacting gut microbiome components and further pinpoint key target mechanisms, as depicted our study flowchart in **Figure 1** (illustration created with biorender.com).

## 2 Materials and methods

### 2.1 Study design

Employing the two-sample Mendelian Randomization (MR) approach, we evaluated the associations between the gut microbiome, osteoclasts, and postmenopausal osteoporosis. This methodology allowed us to explore key genes affecting osteoporosis through osteoclasts, with validation performed using single-cell data. To comprehensively investigate the role of the gut microbiome in postmenopausal osteoporosis, MR analyses were conducted across five different taxonomic levels, including phylum, class, order, family, and genus.

### 2.2 Data sources

#### 2.2.1 Exposure data

The aim of this study was to investigate the association between human genetic variations and the gut microbiome, specifically utilizing single nucleotide polymorphisms (SNPs) associated with the composition of the human gut microbiome as instrumental variables (IVs) in a genome-wide association study (GWAS) dataset. A large-scale multi-ethnic GWAS conducted by the international MiBioGen consortium analyzed genotyping data and 16S ribosomal RNA gene sequencing from 24 cohorts across various countries including Germany, the USA, Denmark, Canada, Israel, Finland, the UK, the Netherlands, Belgium, Sweden, and Korea, comprising a total of 18,340 participants. The study identified 211 taxa, including 9 phyla, 16 classes, 20 orders, 35 families, and 131 genera (12). Gene-related cis-eQTL data were sourced from the eQTLGen Consortium, encompassing data from 31,684 samples (13).

#### 2.2.2 Outcome data 1 - osteoclasts

Osteoclast outcome data were obtained from the GEFOS consortium, a large international collaboration. An eQTL analysis was conducted on 158 independent human osteoclast-like cell cultures derived from peripheral blood mononuclear cells, collected from female patients at Sir Charles Gairdner Hospital in Western Australia. These participants, aged 30 to 70 years and of European descent, underwent dual-energy X-ray absorptiometry bone density measurement. After genotype imputation and quality control, data for 5,373,348 variants were available (14).

#### 2.2.3 Outcome data 2 - postmenopausal osteoporosis

The FinnGen consortium provided GWAS summary statistics for postmenopausal osteoporosis (finn-b-OSTPOPATFRCTURE_POSTEMENO). A binary classification GWAS for postmenopausal osteoporosis among European females were conducted, including 621 cases and 122,861 controls, analyzing 16,379,783 SNPs (https://gwas.mrcieu.ac.uk/datasets/finn-b-OSTPOPATFRCTURE_POSTEMENO/).

### 2.2.4 Single-cell data

Single-cell data were derived from GSE147287, focusing on a human osteoporosis sample (15). Cells expressing more than 200 but fewer than 2,500 genes were identified. A cutoff for mitochondrial genes at 10% and erythrocyte genes at 3% was applied for further filtration. After identifying highly variable genes for analysis, the number of principal components (PCs) was adjusted for different cell clusters before their visualization and annotation. Based on osteoclast-related markers (CDH11 and COL1A1), the data were categorized into osteoclast and non-osteoclast cells to verify the primary distribution of key genes in cells. **Table 1** provides detailed information on the data sources and statistical summaries used in this study. Since this research was based on previously published GWAS summary data, Institutional Review Board approval was not required, and informed consent was obtained from all participants in advance.

**Table 1:** Summary of data sources.

GWAS data					
Traits	Consortium	Year	Population	Sample size	PubMed ID or URL of original
Exposure					
Gut microbiota	MiBioGen	2021	European	18340	33462485
cis-eQTL	eQTLGen	2018	NA	31,684	34475573
Outcome					
Osteoclast	GEFOS	2021	European	158	2947397
Postmenopausal osteoporosis	FinnGen	2021	European	123,482	https://gwas.mrcieu.ac.uk/
scRNA data	Source	Year	Sample	Count of cells	PMID
GSE147287	GEO	2023	1	8084	37105556

### 2.3 Instrumental variable selection

Instrumental variables (IVs) selection for MR analyses followed these criteria: (1) Potential IVs were SNPs significantly associated with each taxa at a genome-wide significance threshold (P < 5.0 × 10^-6); (2) Linkage disequilibrium (LD) between SNPs was calculated using the 1000 Genomes Project European sample as a reference panel, retaining the SNP with the lowest P-value in instances where R^2 < 0.001 (clumping window size = 10,000kb); (3) SNPs with a minor allele frequency (MAF) ≤ 0.01 were excluded; (4) For palindromic SNPs, allele frequency information was used to infer the forward strand alleles (16).

### 2.4 Identification of pleiotropic genes

To delve into the potential mechanisms involved in postmenopausal osteoporosis related to the gut microbiome, a characterization analysis of pleiotropic genes was conducted on identified key loci. Initially, intersections between positive results for gut microbiome-osteoclasts and gut microbiome-postmenopausal osteoporosis were performed to identify common gut microbiota involved. SNPs of these microbiota were analyzed using Multi-marker Analysis of Genome-wide Association Studies (MAGMA v1.07b) to perform gene-based analyses (17). This integrated SNP-level associations from GWAS summary statistics into single gene-level association signals. Key loci corresponding genes were identified, providing insights into the specific processes through which gut microbiota influence osteoporosis via osteoclasts.

### 2.5 Statistical analysis

For characteristics with various IVs, we employed six popular MR methods including Inverse Variance Weighted (IVW), Weighted Median, Simple Mode, MR-Egger regression, Weighted Median Estimator (WME), and MR-PRESSO. The IVW method is mentioned as marginally more effective under certain conditions. Thus, outcomes with multiple IVs are primarily based on the IVW method, with the other five methods serving as supplementary analyses. The principles for MR method selection are: (1) IVW estimation is preferred in the absence of heterogeneity and pleiotropy; (2) the WME method’s results are prioritized when there is heterogeneity but no pleiotropy; (3) when pleiotropy is present, results from MR-Egger method are prioritized (18). Leave-one-out sensitivity analysis involves sequentially removing each SNP to calculate the meta-effect of the remaining SNPs and observing if results change significantly upon the removal of any SNP. Cochran’s Q statistic and the two-sample MR package were used for heterogeneity tests, eliminating IVs with F statistics <10 to minimize weak instrument bias. All statistical analyses were performed using R version 4.2.2, with MR analyses conducted using the TwoSampleMR (version 0.5.6) and MR-PRESSO (version 1.0) packages among others (19).

## 3 Results

### 3.1 Causal influence of gut microbiota on osteoclasts

**Figure 2:**
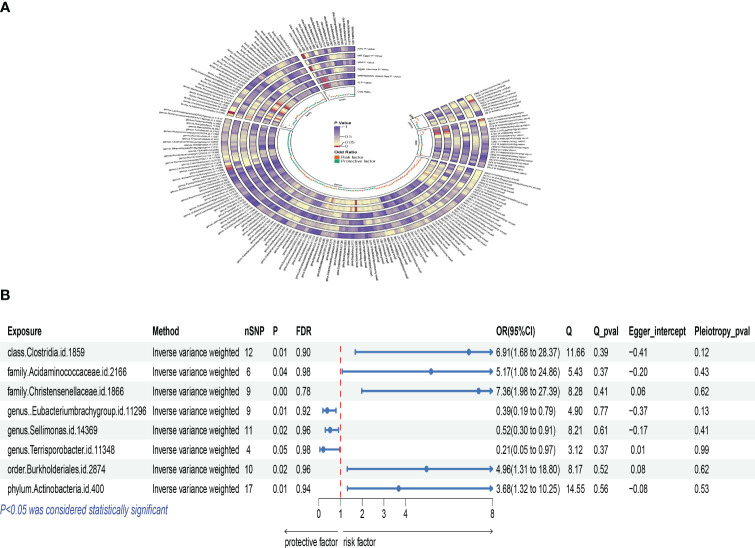
Mendelian analysis results for gut microbiota and osteoclasts; **(A)**: IVW heatmap results of the causal relationship between gut microbiota and osteoclasts; **(B)**: Forest plot results showing the association between gut microbiota and osteoclasts.

Based on Inverse Variance Weighted (IVW) method outcomes, significant positive associations were identified between the genetic predictors of Actinobacteria phylum and osteoclasts, with an odds ratio (OR) of 3.681, 95% confidence interval (CI) of 1.321 to 10.253, and a P-value of 0.013. Similar positive correlations were observed with Clostridia class, Burkholderiales order, Acidaminococcaceae family, and Christensenellaceae family, indicating a complex interplay between gut microbiota and osteoclasts, revealing potential mechanisms of gut microbiota in bone health and disease. No evidence of heterogeneity or pleiotropy was found in these effects (**Supplementary Table S1**). MR-PRESSO global test did not identify any outliers (**Supplementary Table S2**), and detailed scatter plots for each MR analysis are shown in **Supplementary Figure S1**. A heatmap of IVW results for gut microbiota and osteoclasts is presented in **Figure 2A**, with a forest plot for key taxa using the IVW method shown in **Figure 2B**.

### 3.2 Causal effects of gut microbiota on postmenopausal osteoporosis

Using the Inverse Variance Weighted (IVW) method, we explored the relationship between specific gut microbiota and postmenopausal osteoporosis. The Bacillales order showed a negative correlation with postmenopausal osteoporosis accompanied by pathological fractures. The odds ratio (OR) was 0.623, with a 95% confidence interval (CI) of 0.438 to 0.885, and a P-value of 0.008. This suggests that an increased abundance of this microbial group might be associated with a reduced risk of osteoporosis.

**Figure 3:**
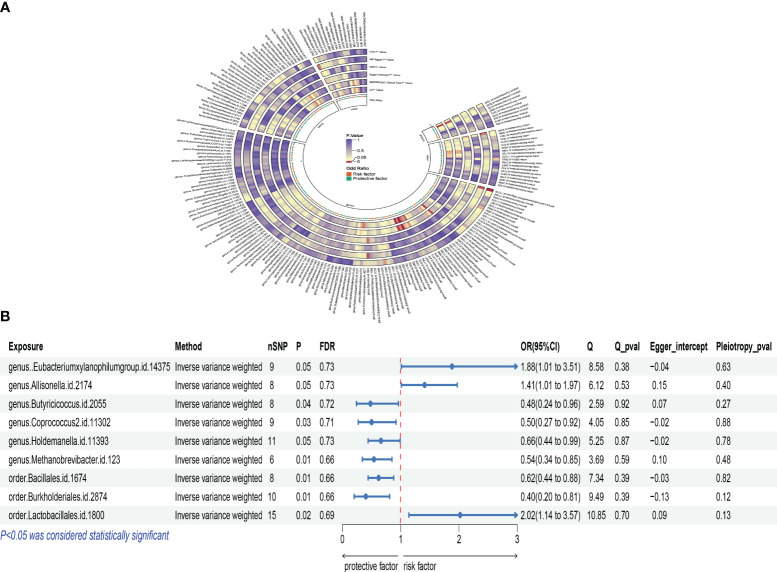
Mendelian analysis results for gut microbiota and postmenopausal osteoporosis; **(A)**: IVW heatmap results of the causal relationship between gut microbiota and postmenopausal osteoporosis; **(B)**: Forest plot results showing the association between gut microbiota and postmenopausal osteoporosis.

Similarly, an increase in the Burkholderiales order was also related to a decreased risk of osteoporosis. The OR was 0.400, with a 95% CI of 0.198 to 0.807, and a P-value of 0.011. Conversely, an increase in the Lactobacillales order was positively correlated with osteoporosis risk. The OR was 2.017, with a 95% CI of 1.141 to 3.565, and a P-value of 0.016. This suggests that an increase in this microbial group might be associated with an increased risk of osteoporosis. Additionally, the Eubacterium xylanophilum group showed a positive correlation, with an OR of 1.884, 95% CI of 1.010 to 3.514, and a P-value of 0.046, indicating that an increase in certain gut microbiota might promote the occurrence of postmenopausal osteoporosis. The increase in the Allisonella genus was related to an increased risk of osteoporosis, with an OR of 1.409, 95% CI of 1.007 to 1.970, and a P-value of 0.045. The Butyricicoccus genus showed a negative correlation with osteoporosis risk, with an OR of 0.478, 95% CI of 0.238 to 0.958, and a P-value of 0.037, suggesting that this microbial group might be beneficial for maintaining bone health. The Coprococcus 2 genus analysis also indicated a negative correlation, with an OR of 0.499, 95% CI of 0.270 to 0.921, and a P-value of 0.026, emphasizing the complex relationship between gut microbiota diversity and the risk of postmenopausal osteoporosis. An increase in the Holdemanella genus might be related to a decreased risk of osteoporosis, with an OR of 0.660, 95% CI of 0.438 to 0.993, and a P-value of 0.046. The analysis of the Methanobrevibacter genus also indicated a negative correlation with osteoporosis risk, with an OR of 0.541, 95% CI of 0.343 to 0.853, and a P-value of 0.008, further supporting the potential protective role of gut microbiota in bone health. According to Cochran’s Q test, there was no evidence of heterogeneity in the effects of specific gut microbiota on PMOP (P > 0.05) (**Supplementary Table S3**). All P-values for the MR-Egger intercept tests were >0.05, indicating no horizontal pleiotropy (**Supplementary Table S3**). Moreover, no outliers were detected in the MR-PRESSO global test (**Supplementary Table S4**). Detailed scatter plots for each MR method’s analysis are shown in **Supplementary Figure S2**. We depicted a heatmap of the IVW results for gut microbiota and PMOP in **Figure 3A** and presented a forest plot for the IVW method in **Figure 3B**.

### 3.3 Identification of key genes

**Figure 4:**
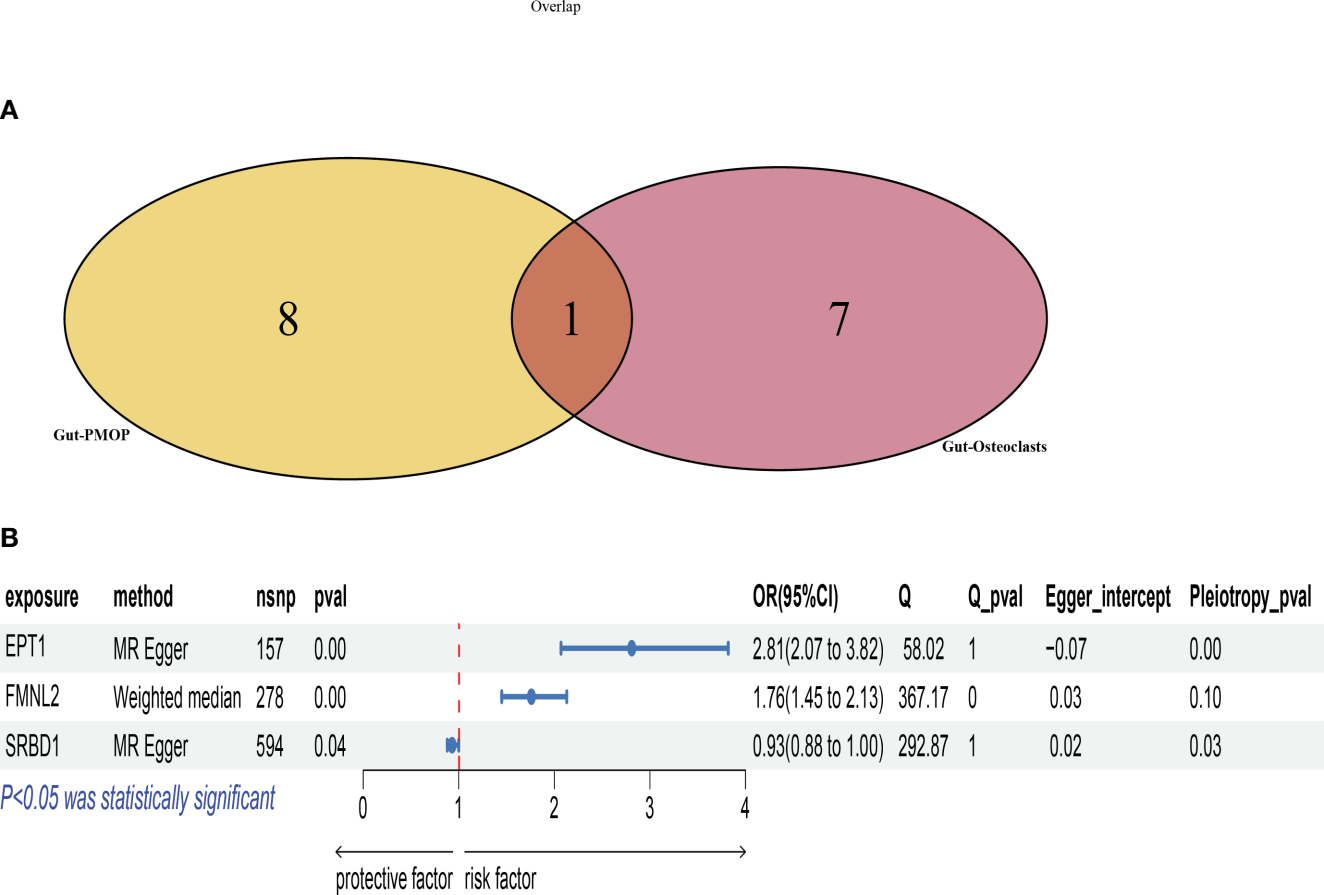
Selection and Mendelian analysis of key genes. **(A)**: Venn diagram intersecting key microbiota for osteoclasts and key microbiota for postmenopausal osteoporosis; **(B)**: Forest plot showing the validation of key genes based on eQTL data.

We intersected the data with positive results for gut microbiota-osteoclasts and gut microbiota-postmenopausal osteoporosis (**Figure 4A**). The selected microbial species for both categories are listed in **Supplementary Table S5**, from which we identified a key microbial order, Burkholderiales.id.2874. This microbe showed significant correlations at the corresponding SNP sites for both groups, with leave-one-out plots presented in **Supplementary Figure S3**. Key pleiotropic genes were identified using the MAGMA software, suggesting these genes are involved in the process by which gut microbiota influences postmenopausal osteoporosis through osteoclasts. A total of four key genes were identified: EPT1, FMNL2, SRBD1, and RGPD6, with the MAGMA analysis results listed in **Supplementary Table S6**.

To further validate the causal relationship between these key genes and postmenopausal osteoporosis, we utilized the SMR software tool with cis-eQTL data from 31,684 samples from the eQTLGen Consortium. Initially, the ENSB numbers for these key genes on chromosomes were retrieved from NCBI, followed by the download of corresponding eQTL data. Unfortunately, eQTL data were only retrieved for three genes: EPT1, FMNL2, and SRBD1, with the specific eQTL data used listed in **Supplementary Tables S7-S9**. We then conducted Mendelian randomization analysis based on these key genes’ eQTL data and the outcomes for postmenopausal osteoporosis, as shown in **Figure 4B**. Scatter plots for each method’s results are presented in **Supplementary Figure S4**.

Among the results, EPT1’s Q_pval was 1, indicating a lack of evidence for heterogeneity. However, its Pleiotropy_pval was very small (8.59E-05), significantly less than 0.05, indicating pleiotropy. FMNL2’s Q_pval was 0.000226263, indicating heterogeneity. Its Pleiotropy_pval was 0.096037321, suggesting insufficient evidence for pleiotropy. SRBD1’s Q_pval was 1, indicating no heterogeneity. Its Pleiotropy_pval was 0.027538112, indicating pleiotropy.

Based on these findings and the three main principles for choosing MR methods, the MR Egger method was prioritized for EPT1 and SRBD1 due to the presence of pleiotropy. The Weighted median method was used for FMNL2 due to heterogeneity but no pleiotropy.

Analysis showed that the EPT1 gene had a significant positive correlation with postmenopausal osteoporosis risk. The P-value was 5.86E-10, with an odds ratio (OR) of 2.810622008 and a 95% confidence interval (CI) from 2.068786205 to 3.818469039. This suggests an increase in EPT1 gene expression might be associated with an increased risk of postmenopausal osteoporosis.

FMNL2 gene analysis using the Weighted median method involved 278 SNPs. This also showed a significant positive correlation, with a P-value of 6.65E-09, an OR of 1.760531588, and a 95% CI from 1.454205367 to 2.131384976. This indicates its increased expression might be related to a higher risk of osteoporosis.

The SRBD1 gene, analyzed using the MR Egger method with 594 SNPs, had a P-value of 0.036986549 and an OR of 0.934467962. This suggests its increased expression might be associated with a reduced risk of osteoporosis.

### 3.4 Validation of key genes using single-cell data

**Figure 5:**
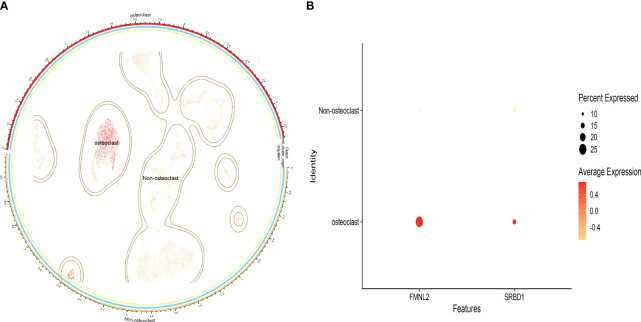
Single-cell data analysis results; **(A)**: Cell distribution map after clustering annotation of the dataset; **(B)**: Bubble chart showing the proportion of key genes across different cells.

By clustering annotation of human osteoporosis single-cell datasets, we manually annotated only the osteoclast clusters using markers CDH11 and COL1A1 (**Supplementary Figure S5**), dividing the data into osteoclast and non-osteoclast clusters (**Figure 5A**). The distribution of the identified key genes within cells was analyzed to validate whether these key genes were predominantly expressed in osteoclasts. Our findings indicated that FMNL2 and SRBD1 were significantly distributed within osteoclast clusters, with FMNL2 exhibiting a higher expression level than SRBD1 (**Figure 5B**). On the other hand, EPT1 was not detected in osteoclast clusters, suggesting it might not be a key gene expressed in osteoclasts, possibly due to the selection criteria applied during cell filtering.

## 4 Discussion

In our study, we have deepened the understanding of the gut-bone axis concept, which refers to how gut microbiota can indirectly influence bone health by affecting the behavior of osteoclasts. We discovered that certain gut microbial communities, such as Actinobacteria and Clostridia, are positively correlated with an increase in osteoclasts, suggesting potential mechanisms through which gut microbiota might influence bone metabolism by regulating inflammatory responses or hormone levels. This finding echoes previous research that also reported associations between changes in the gut microbiome and osteoporosis. Moreover, through causal analysis of the impact of gut microbiota on postmenopausal osteoporosis, we identified several microbial groups associated with reduced risk of osteoporosis, such as Bacillales and Burkholderiales. These results lay a theoretical foundation for developing interventions targeting specific gut microbiota, potentially reducing the risk of osteoporosis by regulating the abundance of these beneficial microbial communities. Furthermore, our research has identified several key genes (FMNL2 and SRBD1) whose expression in osteoclasts is significantly associated with the risk of postmenopausal osteoporosis. These genes may function by influencing the differentiation and activation of osteoclasts.

The gut microbiota and postmenopausal osteoporosis have always been topics of interest in the medical community. Postmenopausal osteoporosis is a type of bone disease characterized by decreased bone mass and the destruction of bone microarchitecture, caused by estrogen deficiency, which can lead to an increased incidence of fragility fractures. In recent years, an increasing number of studies have begun to focus on the relationship between the gut microbiota and postmenopausal osteoporosis. He et al. found significant differences in the composition of the gut microbiota between patients with osteoporosis and osteopenia compared to those with normal bone density among 106 postmenopausal women, suggesting a link between the gut microbiota and reduced bone density (20). Xu et al. (2017) proposed the gut microbiota as a potential new target for the treatment of postmenopausal osteoporosis, highlighting that the gut microbiota can influence bone metabolism through various mechanisms, such as regulating the host’s immune system, particularly affecting inflammation and autoimmune responses. For example, the gut microbiota can promote the production of regulatory T cells (Treg) by producing short-chain fatty acids (SCFAs) such as butyrate and propionate, thereby exerting anti-inflammatory effects (21). On the other hand, the gut-bone axis refers to the interaction between the gut and bones, where the gut microbiota can directly or indirectly influence bone metabolism. SCFAs produced by the gut microbiota are considered key mediators in the gut-bone axis, capable of promoting calcium absorption and regulating the production of hormones related to bone metabolism, such as stimulating the secretion of insulin-like growth factor 1 (IGF-1), thus affecting bone formation and resorption (20). Gut microbes can metabolize the food components ingested by the host, producing a range of metabolites that can be beneficial or harmful to the host. In addition to SCFAs, the gut microbiota can also produce other substances with potential impacts on bone metabolism, such as biotin and vitamin K, which are essential for maintaining normal bone metabolism (22).

In clinical practice, osteoclasts are closely related to postmenopausal osteoporosis. Several therapeutic drugs targeting osteoclasts have been developed for the treatment of postmenopausal osteoporosis. Research indicates that the excessive activation of osteoclasts is one of the main causes of postmenopausal osteoporosis (23). Therefore, inhibiting the differentiation and activity of osteoclasts has become an important strategy in the treatment of postmenopausal osteoporosis. Osteoclasts are the primary cell type involved in bone resorption, and their activity is regulated by various factors, including cytokines and hormones (24). Particularly, an imbalance in the RANKL (Receptor Activator of Nuclear factor Kappa-B Ligand) and OPG (Osteoprotegerin) system is considered a key mechanism for the increased activity of osteoclasts. RANKL can promote the differentiation and activation of osteoclasts, while OPG can act as a “decoy” receptor for RANKL, reducing its effect on its receptor RANK, thereby inhibiting the activity of osteoclasts (24). This association may also be driven by the common pathogenesis or metabolic interactions between the two diseases. By focusing on the gut microbiota, we identified a microbial community that affects both: order Burkholderiales.id.2874. In previous studies, a causal relationship between Burkholderiales and lumbar spine fractures has been identified, corroborating our findings that this genus indeed has an unknown connection with bone (25). Building on prior research, we expanded the related mechanisms by incorporating the impact on osteoclasts and predicting their potential downstream targets. On the other hand, our results show that Burkholderiales promotes an increase in osteoclasts while simultaneously reducing the risk of osteoporosis. Regarding this contradiction, we believe the reasons may include the following points: Immune Regulation: Burkholderiales may influence bone metabolism by modulating the host’s immune response. Impact of Metabolites: Gut microbes, including Burkholderiales, can produce various metabolites such as short-chain fatty acids (SCFAs) (26). These metabolites are known to influence bone metabolism through multiple pathways, including enhancing bone density by regulating calcium absorption and hormone balance. Complexity of Microbiota Composition: The gut microbiota is a complex ecosystem, and the effects of a single microbial order may be modulated by other microbial community members. Most importantly, we believe that while an increase in osteoclasts is typically associated with osteoporosis, bone remodeling is a dynamic process involving the activities of both osteoclasts and osteoblasts. In some cases, Burkholderiales may simultaneously activate signals related to osteogenesis, counterbalancing the negative effects of osteoclastic activity. However, as the GWAS database lacks relevant osteoblast data, we cannot verify whether this bacterium affects osteoblasts similarly. This influence may offset or exceed the generation of osteoclasts, ultimately leading to the phenotype of osteoporosis, highlighting a limitation of this study. Research by Cao et al. found that certain gut microbiota, including the Burkholderiales order, are associated with bone density changes in autoimmune diseases, suggesting a possible link with osteoporosis (27). A comprehensive Mendelian randomization study by Luo et al. (2023) observed a connection between Burkholderiales and lumbar spine pelvic bone density. Their findings suggest that certain gut bacteria, including Burkholderiales, may enhance osteoblast function and inhibit osteoclast activity (25). Burkholderiales and Methanobrevibacter significantly affect the growth and metabolic potential of their fungal hosts by altering host metabolic pathways and inhibiting fungal cell invasion mechanisms (28). Butyricicoccus, a butyrate-producing bacterium, has been shown to improve the composition of the gut microbiota and enhance resistance through various metabolic pathways, impacting host health (29). Specific strains of Lactobacillus reuteri from the Lactobacillales order demonstrate host-specific adaptations by affecting the composition of the gut microbiota and influencing host immune responses (30). Guo et al. explored how dysbiosis of the gut microbiota is related to estrogen levels and may lead to osteoporosis (31). They noted that changes in the microbiota, including shifts in Burkholderiales, could lead to alterations in osteoclast activity. These studies, consistent with our findings, further illustrate the potential of order Burkholderiales.id.2874 to affect postmenopausal osteoporosis by influencing osteoblast function. Wei et al.’s research found that changes in the gut microbiota, including the Lactobacillus genus, are associated with osteoporosis. These findings are consistent with our research (32). Bacteria of the Butyricicoccus genus can produce short-chain fatty acids (SCFAs), such as butyrate, which are crucial for gut health and anti-inflammatory responses. Butyrate may have a positive impact on bone health by reducing inflammation and oxidative stress (33). Additionally, Wang et al.’s research found that changes in the gut microbiota associated with osteoporosis are related to amino acid metabolic pathways. Studies suggest that dysbiosis of the gut microbiota and its impact on amino acid metabolism could be targets for osteoporosis intervention (34). These findings are also consistent with our research. In our study, we discovered significant correlations between specific gut microbiota and dysfunction of osteoclasts as well as the risk of postmenopausal osteoporosis. Metabolites and bioactive factors produced by bacteria may regulate host metabolism through multiple indirect and interdependent steps, thereby affecting bone metabolism. For example, Dong’s research reported that microbiota-derived metabolites play a crucial role in dynamic interactions, particularly in bone homeostasis. In this sense, SCFAs, including acetate, propionate, and butyrate, indirectly promote bone formation by regulating insulin-like growth factor 1 (IGF-1) (35).

To further explore the potential mechanisms involved in this process, we conducted MAGMA analyses and single-cell data validation, identifying two key genes, FMNL2 and SRBD1, that may play roles in these mechanisms. FMNL2 (Formin-like 2) is a protein that regulates cytoskeletal reorganization, affecting cell migration and morphology (36). In the skeletal system, the dynamic balance between osteoclasts and osteoblasts determines bone health. FMNL2, by influencing cell polarity and migration capability, may indirectly affect the processes of bone resorption and formation. Currently, there is limited research on SRBD1 (S1 RNA binding domain 1) in relation to bone. Most studies on SRBD1 focus on its role in yeast, its function in the growth processes of specific plants, and its involvement in human diseases, especially its association with ocular diseases such as glaucoma (37). In summary, research on the genetic functions and treatments of osteoporosis provides new insights and potential therapeutic targets. Understanding how these signaling pathways and genes affect the balance between bone resorption and formation offers important information for developing new treatment strategies. Particularly, future studies on genes like FMNL2 may reveal their specific roles and therapeutic potential in osteoporosis. Despite some important discoveries, our research has limitations. For example, while Mendelian randomization analysis can reduce the impact of confounding factors, it relies on strong instrumental variables. Our study may also be influenced by some confounding factors, such as bacterial metabolites and bioactive factors affecting bone metabolism through various indirect pathways. This is one of the limitations of this study, as it is difficult to control for related confounding factors in Mendelian studies compared to clinical individual data. Moreover, while single-cell data provide detailed information on gene expression, the relatively small sample size may limit the generalizability of our findings. Future research should continue to explore the specific interactions between the gut microbiome and bone health, especially how it can regulate osteoporosis by affecting osteoclast behavior, to provide more precise therapeutic targets for the treatment of this disease. Although this study primarily focused on bacteria, particularly the role of the Burkholderiales order, viruses and fungi can also have significant impacts on host bone metabolism and health. This is a limitation of the present study. To obtain a more comprehensive picture of the relationship between the gut microbiome and bone health, the study of viruses and fungi should not be overlooked. We plan to use metagenomic and metatranscriptomic sequencing techniques in future research to analyze the composition of viruses and fungi in the gut microbiome.

## 5 Conclusion

This study utilizing the two-sample Mendelian Randomization method, delved deeply into the relationship between the gut microbiome, osteoclasts, and postmenopausal osteoporosis, particularly highlighting the significant impact of microbial communities such as the Burkholderiales order on osteoclasts and postmenopausal osteoporosis. The analysis identified two key genes, FMNL2 and SRBD1, as playing pivotal roles, and found that the gut microbiome indirectly influences osteoporosis by regulating these genes. Additionally, it pointed out a significant correlation between the Burkholderiales order and a reduced risk of osteoporosis. These findings not only deepen our understanding of the gut-bone axis concept but also provide new therapeutic strategies through the modulation of the gut microbiome composition or targeted interventions on specific genes to reduce the risk of postmenopausal osteoporosis.

## Data availability statement

The datasets presented in this study can be found in online repositories. The names of the repository/repositories and accession number(s) can be found in the article/**Supplementary Material**.

## Ethics statement

Ethical review and approval were not required for the study on human participants in accordance with the local legislation and institutional requirements. Written informed consent for participation was not required for this study in accordance with the national legislation and the institutional requirements.

## Author contributions

HX: Conceptualization, Formal analysis, Project administration, Writing – original draft, Writing – review & editing, Data curation, Methodology, Supervision, Validation. YW: Data curation, Validation, Writing – review & editing, Methodology. YC: Methodology, Validation, Writing – review & editing, Conceptualization, Resources. RC: Methodology, Writing – review & editing, Formal analysis, Software. CY: Writing – review & editing, Conceptualization, Data curation, Supervision. BG: Conceptualization, Writing – review & editing, Project administration, Validation. YX: Conceptualization, Validation, Writing – review & editing, Data curation, Formal analysis, Resources.
