# Supplementary material for: Gut-bone axis research: unveiling the impact of gut microbiota on postmenopausal osteoporosis and osteoclasts through Mendelian randomization
Source: Front Endocrinol (Lausanne). 2024 May 31;15:1419566. doi: 10.3389/fendo.2024.1419566 (PMC11176613; doi:10.3389/fendo.2024.1419566)

Supplementary Material for Figure

Supplementary Figure S1

Causal effects of the gut microbiome on osteoclasts. The figure displays detailed scatter plots of positive results for different microbes analyzed by each MR method. The microbes results shown in Supplementary Figures A-I are respectively: genus Eubacterium xylanophilum group id.14375; genus Allisonella id.2174; genus Butyricicoccus id.2055; genus Coprococcus2 id.11302; genus Holdemanella id.11393; genus Methanobrevibacter id.123; order Bacillales id.1674 scatter; order Burkholderiales id.2874 scatter; order Lactobacillales id.1800 scatter.

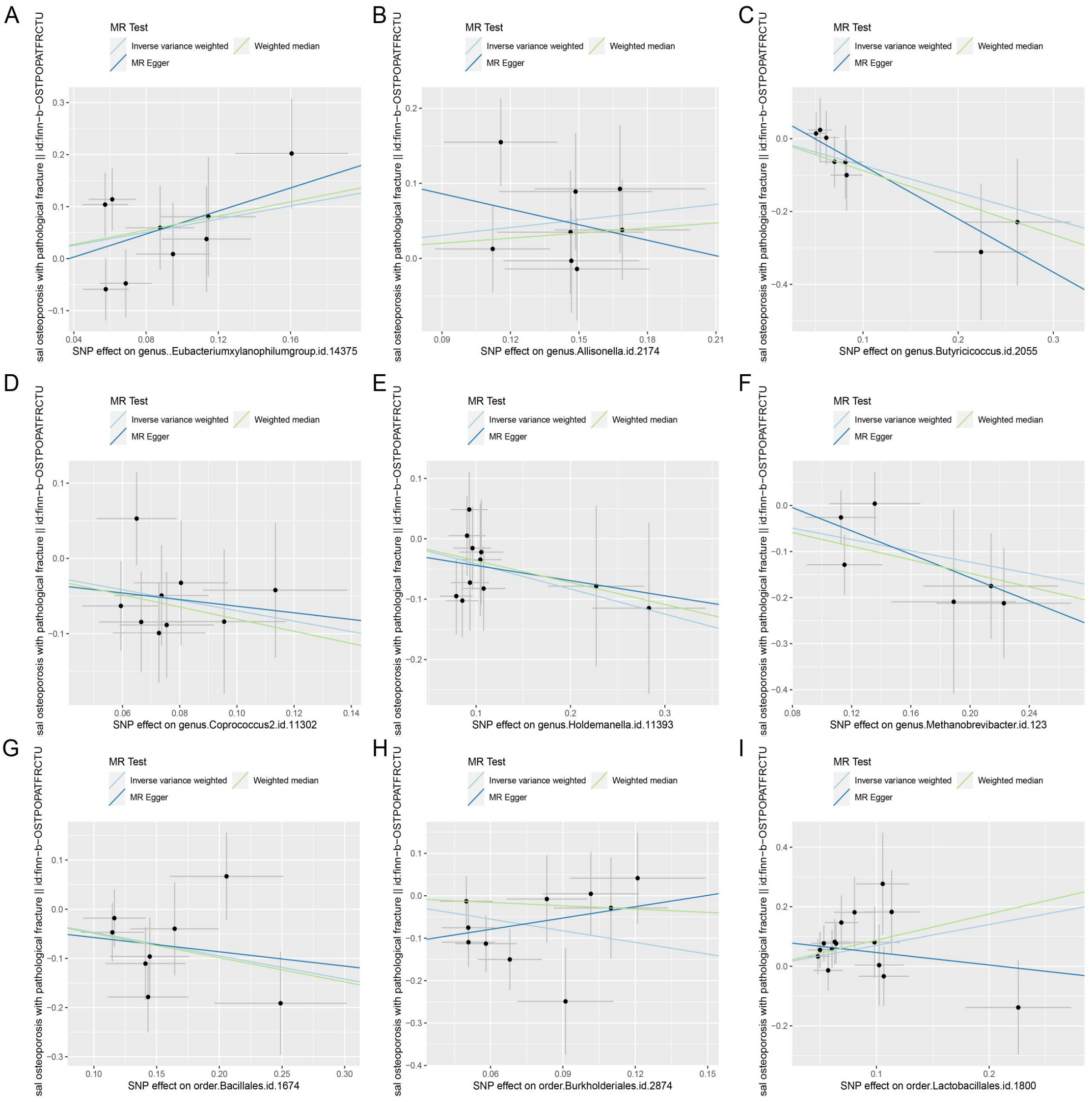

Supplementary Figure 2

Causal effects of the gut microbiome on postmenopausal osteoporosis. The figure displays detailed scatter plots of positive results for different microbes analyzed by each MR method. The microbes results shown in Supplementary Figures A-H are respectively: class Clostridia id.1859 scatter; family Acidaminococcaceae id.2166 scatter; family Christensenellaceae id.1866 scatter; genus Eubacterium brachy group id.11296 scatter; genus Sellimonas id.14369 scatter; genus Terrisporobacter id.11348 scatter; order Burkholderiales id.2874 scatter; phylum Actinobacteria id.400 scatter.

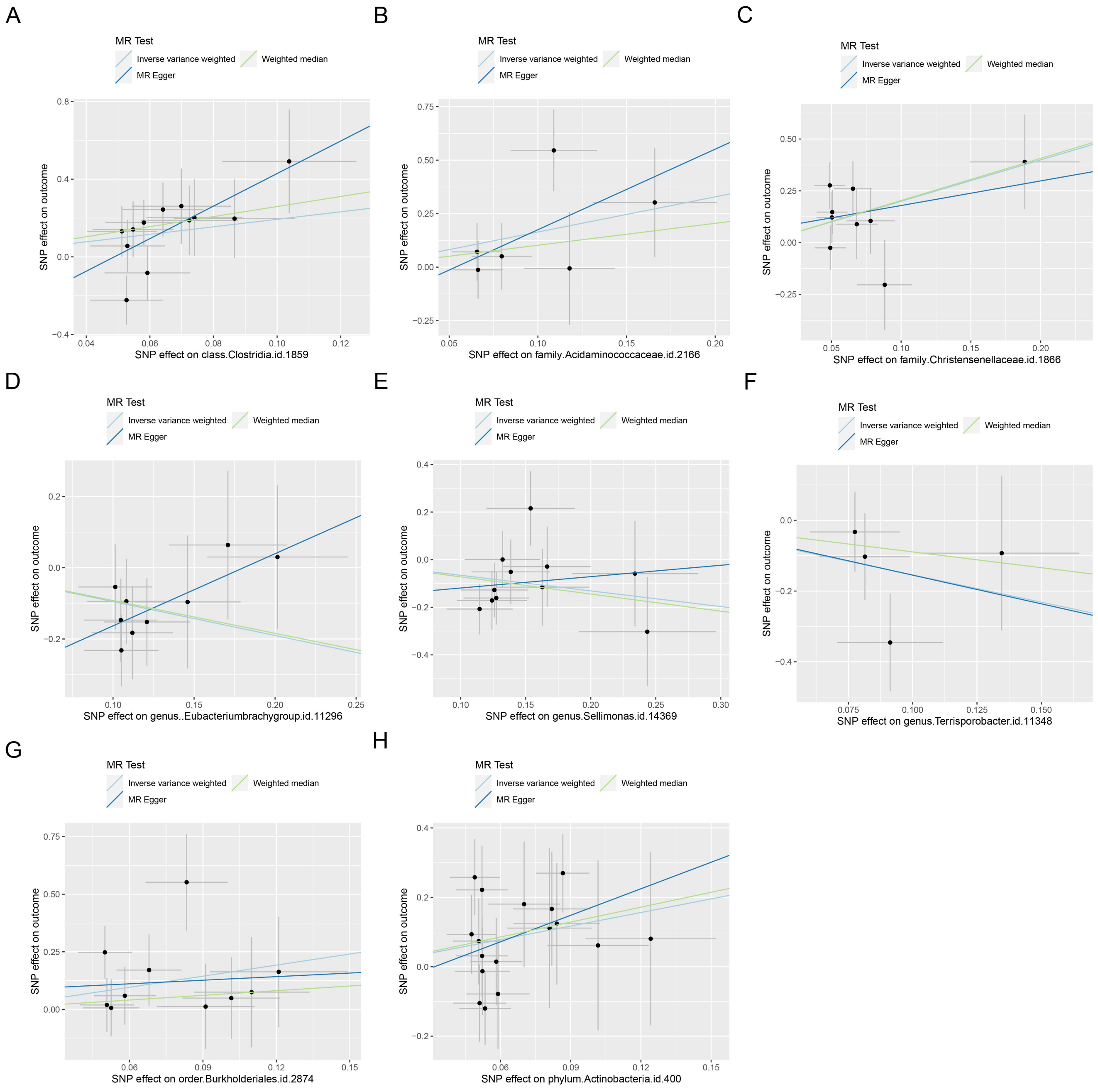

Supplementary Figure S3

Key microbial order Burkholderiales id.2874, with the corresponding SNP loci leave-one-out plots in two groups depicted as: a: order Burkholderiales id.2874 as the exposure, with the outcome being postmenopausal osteoporosis shown in the leave-one-out result plot; B: order Burkholderiales id.2874 as the exposure, with the outcome being osteoclasts shown in the leave-one-out result plot.

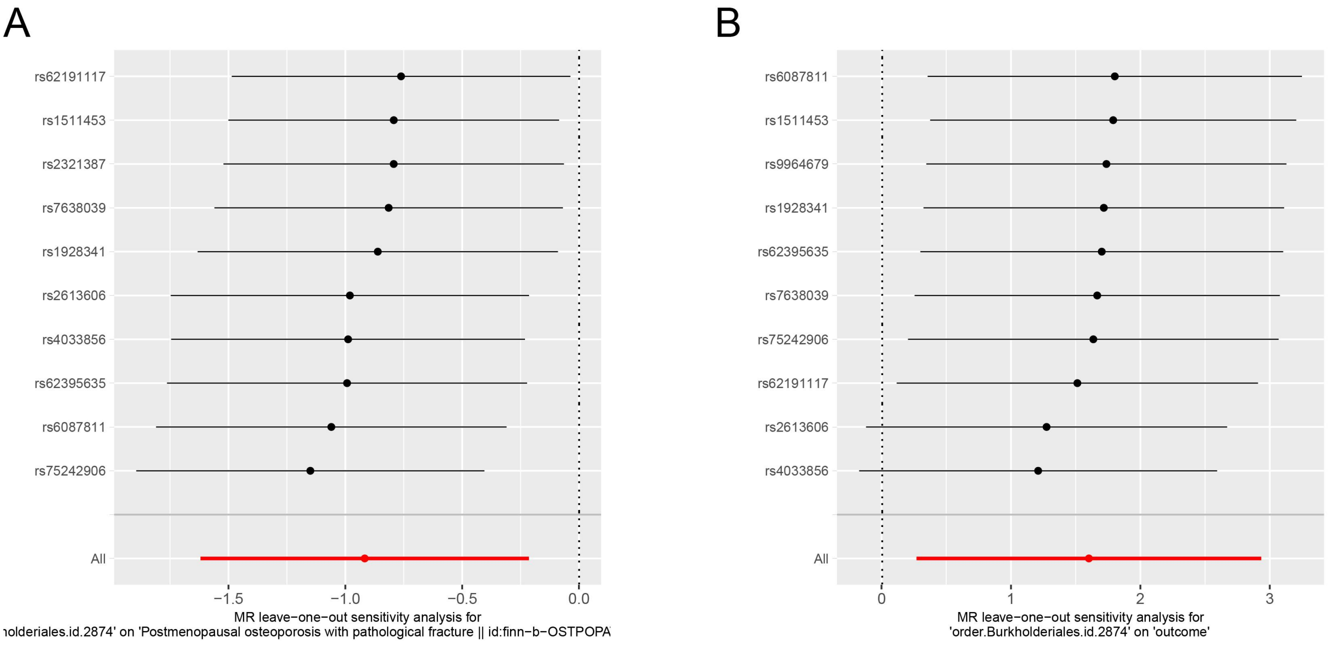

Supplementary Figure S4

The scatter plots of the results from the Mendelian randomization analysis of eQTL data for key genes and postmenopausal osteoporosis. Panels A-C respectively display the results for EPT1; FMNL2; SRBD1.

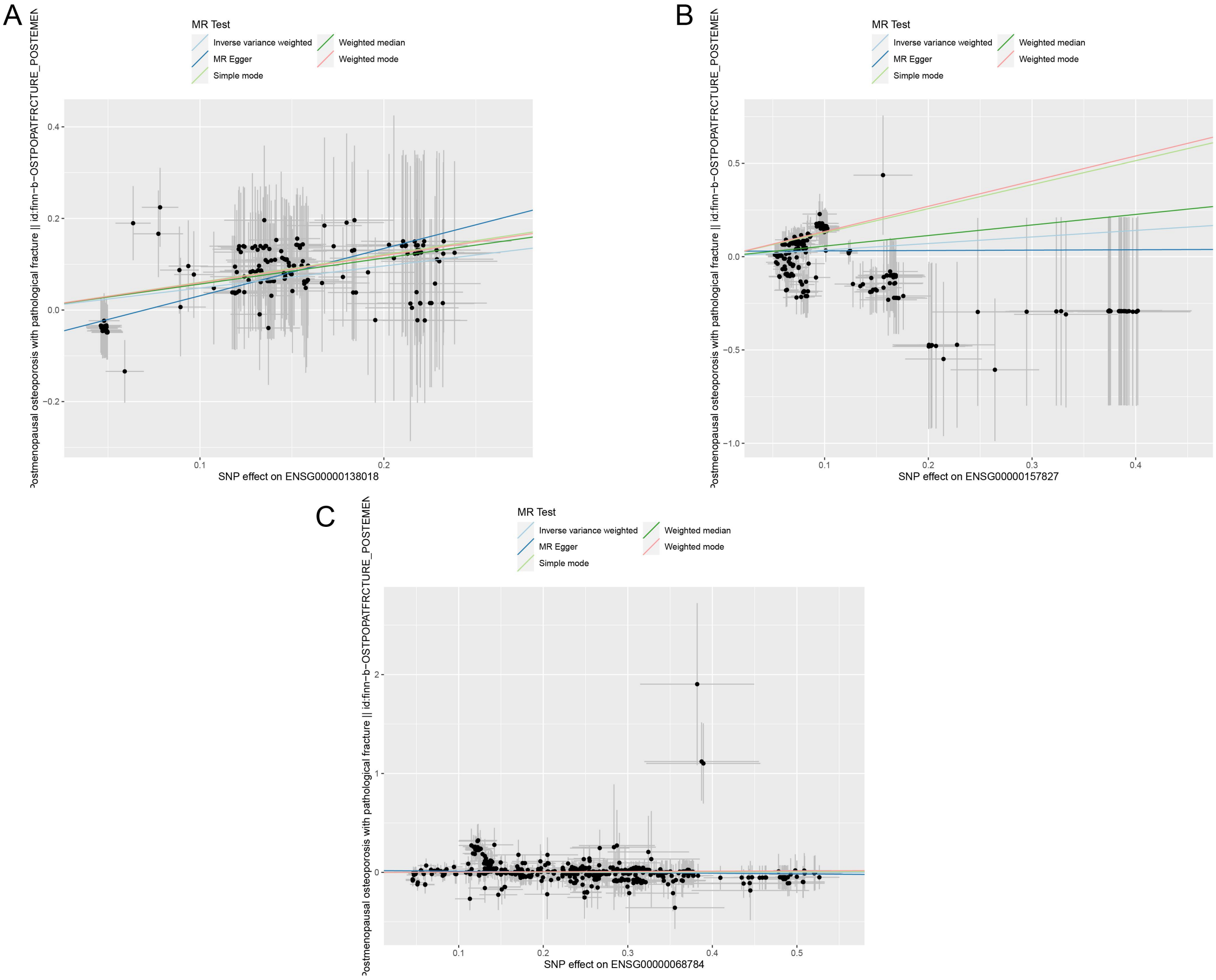

Supplementary Figure S5

Based on markers CDH11 and COL1A1, osteoclast clusters were manually annotated, revealing that key markers are primarily distributed in clusters 0,10and 11. These two clusters have been named as osteoclast clusters.

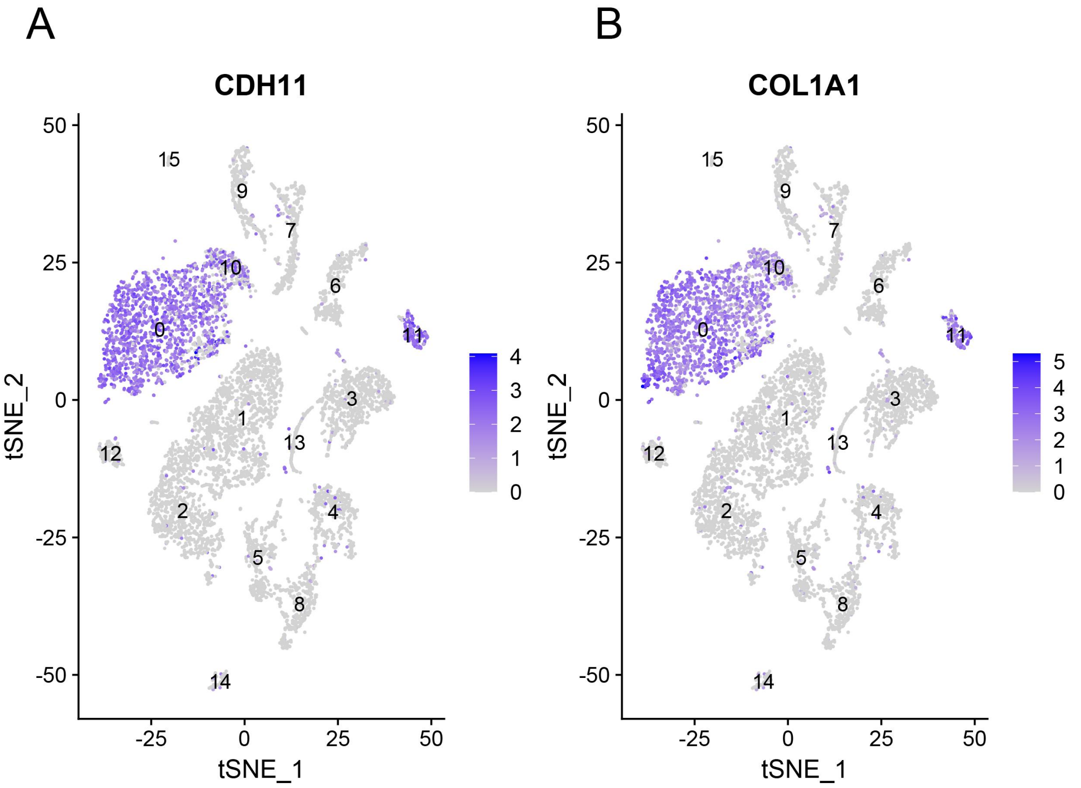

Supplement: Supplementary file 1 [file DataSheet_1.pdf]
